# Regional Variation in Parasite Species Richness and Abundance in the Introduced Range of the Invasive Lionfish, *Pterois volitans*

**DOI:** 10.1371/journal.pone.0131075

**Published:** 2015-06-22

**Authors:** Andrew J. Sellers, Gregory M. Ruiz, Brian Leung, Mark E. Torchin

**Affiliations:** 1 Smithsonian Tropical Research Institute, Apartado 0843–03092, Balboa, Ancon, Panama, Republic of Panama; 2 Department of Biology, McGill University, Montreal, Québec, Canada; 3 Smithsonian Environmental Research Center, Edgewater, Maryland, United States of America; California Polytechnic State University, UNITED STATES

## Abstract

Parasites can play an important role in biological invasions. While introduced species often lose parasites from their native range, they can also accumulate novel parasites in their new range. The accumulation of parasites by introduced species likely varies spatially, and more parasites may shift to new hosts where parasite diversity is high. Considering that parasitism and disease are generally more prevalent at lower latitudes, the accumulation of parasites by introduced hosts may be greater in tropical regions. The Indo-Pacific lionfish (*Pterois volitans*) has become widely distributed across the Western Atlantic. In this study, we compared parasitism across thirteen locations in four regions, spanning seventeen degrees of latitude in the lionfish's introduced range to examine potential spatial variation in parasitism. In addition, as an initial step to explore how indirect effects of parasitism might influence interactions between lionfish and ecologically similar native hosts, we also compared parasitism in lionfish and two co-occurring native fish species, the graysby grouper, *Cephalopholis cruentata*, and the lizardfish, *Synodus intermedius*, in the southernmost region, Panama. Our results show that accumulation of native parasites on lionfish varies across broad spatial scales, and that colonization by ectoparasites was highest in Panama, relative to the other study sites. Endoparasite richness and abundance, on the other hand, were highest in Belize where lionfish were infected by twice as many endoparasite species as lionfish in other regions. The prevalence of all but two parasite species infecting lionfish was below 25%, and we did not detect an association between parasite abundance and host condition, suggesting a limited direct effect of parasites on lionfish, even where parasitism was highest. Further, parasite species richness and abundance were significantly higher in both native fishes compared to lionfish, and parasite abundance was negatively associated with the condition index of the native grouper but not that of the lionfish or lizardfish. While two co-occurring native fishes were more heavily parasitized compared to lionfish in Panama any indirect benefits of differential parasitism requires further investigation. Future parasitological surveys of lionfish across the eastern coast of North America and the Lesser Antilles would further resolve geographic patterns of parasitism in invasive lionfish.

## Introduction

Introduced species can alter population-to-ecosystem level processes and cause economic damage [1–3]. Their impacts are determined, in part, by their demographic success (abundance and spread) in the invaded region [4]. Natural enemies, including parasites, can limit the demographic performance of invaders [5–10]. Parasites can exert strong negative effects on hosts [11–13] and affect invasion dynamics of introduced species [5, 14, 15]. Previous research on the role of parasites in biological invasions demonstrates that introduced species often escape parasites from their native range but can also accumulate novel parasites in their new range [5, 15, 16, 17]. Since parasite species richness and prevalence often exhibit strong spatial variation across a host's range [18, 19], the accumulation of parasites by introduced hosts is likely to also vary across broad geographic scales.

Spatial variation in parasitism among host populations can be associated with the age of the population [20], host density and size [21], as well as the occurrence of alternate hosts [22]. There can also be strong geographic patterns for some parasite communities [23]. In a recent review, Schemske *et al*. [24] suggest that parasite richness and prevalence increase at low tropical latitudes, where diverse host assemblages may support a greater overall diversity of parasites [25, 26]. If parasite diversity is indeed greater at low latitudes, there may be a greater potential for native parasites to shift to use novel introduced hosts at lower latitudes than higher latitudes. While variation in patterns of parasitism is interesting, the associated ecological consequences may be best understood in a community context. In particular, differential parasitism in native versus introduced hosts can potentially alter competitive interactions [15]. Differential parasitism may arise because introduced hosts escape their native parasites, and accumulate few new ones in their introduced range [5, 15, 16, 17]. Such differences in parasitism can give introduced hosts a competitive advantage over native competitors [15].

In this study, we examine regional variation in parasitism in the invasive Indo-Pacific red lionfish (*Pterois volitans*), and begin to explore possible differences in parasitism between lionfish and native competitors. Lionfish were probably introduced by aquarium releases as early as the 1980s in Florida, representing the first known lionfish population established in the Atlantic Ocean [27]. They have since spread rapidly over a large area along the southeastern United States, the Gulf of Mexico, and throughout the entire Caribbean, where they can have considerable direct and indirect impacts on native communities [28]. Lionfish likely compete for prey with native meso-predators [28, 29], but it is unclear whether parasites might indirectly influence these interactions.

A number of recent studies provide new records of parasites in invasive lionfish [30–32]; however, only one compared parasite richness and abundance across multiple sites [32], and none have compared parasitism broadly across multiple regions in its extensive introduced range. Thus, we examine regional variation in parasite species richness and abundance in the introduced range of the lionfish across thirteen sites in Florida, Mexico, Belize and Panama. We hypothesize that parasite species richness and abundance varies spatially among lionfish populations, but that introduced lionfish will have likely accumulated more native parasite species in the southernmost tropical regions, relative to other study sites. Further, as an initial step to evaluate the potential for parasite-mediated interactions between lionfish and native competitors, we compare parasitism between lionfish, the graysby grouper *Cephalopholis cruentata*, and the lizardfish, *Synodus intermedius* in the southernmost region, Panama, where we observed the highest ectoparasite richness on lionfish. We hypothesize that parasitism is greater and has a stronger negative effect on the condition index of native fish species, relative to lionfish.

## Methods

### Ethics Statement

Fish collections were carried out in accordance with guidelines for the humane treatment of vertebrates established by McGill University and the Smithsonian Institution. Collection protocols were approved by McGill University's Animal Care Committee (protocol number 2013–7371) and the Smithsonian Tropical Research Institute (protocol number 2013-0330-2016). To avoid contact with lionfish's venomous spines, fish were collected using a pole spear. In most cases this method resulted in immediate death; however, in the event that a fish was not killed by the spear, the spinal cord was severed to eliminate unnecessary suffering. Field collection permits were issued by the Florida Fish and Wildlife Conservation Commission, Belize Fisheries Department, Mexico's Comisión Nacional de Areas Naturales Protegidas, Panama's Autoridad Nacional del Ambiente, and Autoridad de Recursos Acuáticos de Panamá. All native fish were collected outside protected areas. Lionfish were in some cases collected from protected areas with permission from local authorities (Panama: Galeta Point; Mexico: Contoy Island National Park, Xcalak National Park).

### Regional comparison of lionfish parasites

In order to compare parasitism in lionfish across their introduced range, we collected lionfish at thirteen reef sites in four regions spread across seventeen degrees of latitude in the Western Atlantic (see Table 1 for coordinates), and examined them for metazoan ecto- and endoparasites. All sites consisted of rocky or coral reefs with depths ranging between 12 and 30 meters, with the exception of Galeta Point, Panama, which was a shallow (0–5 m) back reef lagoon. We grouped these sites into four different regions (Florida, Mexico, Belize, and Panama), with two to four sites per region. To maximize the independence of our sites (reefs), we ensured that these were separated by at least 2 Km, the maximum recorded displacement distance for *P*. *volitans* after larval settlement [28]. In other words, post-settlement lionfish are unlikely to move between our sampling sites unaided. We did not statistically remove spatial autocorrelation, because latitudinal trends are by definition spatially oriented. Thus, removing spatial autocorrelation would remove potential latitudinal patterns of interest.

**Table 1:** Date, coordinates, and collection effort for each collection site in four regions for latitudinal survey of *Pterois volitans* parasites.

Region	Site	Date	Coordinates	N
Florida, USA	Jupiter	May-2011	26.93°N 79.98°W	27
Florida, USA	Keys	May-2011	24.74°N 80.80°W	17
Yucatan, Mexico	Contoy Island	Apr-2012	21.52°N 86.80°W	20
Yucatan, Mexico	Akumal	Apr-2012	20.34°N 87.34°W	20
Yucatan, Mexico	Xkalac	Apr-2012	18.27°N 87.82°W	20
Belize	Tobacco Cay	Nov-2011	16.89°N 88.06°W	20
Belize	Carrie Bow Cay	Nov-2011	16.80°N 88.08°W	20
Belize	Curlew Cay	Nov-2011	16.78°N 88.07°W	22
Belize	South Cut	Nov-2011	16.70°N 88.08°W	20
Panama	Portobelo	Oct-2011	9.55°N 79.68°W	23
Panama	Naranjo Island	Sep-2011	9.43°N 79.80°W	21
Panama	Galeta Point	Feb-2011	9.40°N 79.86°W	23
Panama	Hospital Point	Apr-2011	9.33°N 82.22°W	20

All lionfish were collected using a pole spear and SCUBA equipment (see Ethics Statement), and kept in a mesh bag for the duration of the dive. At the surface they were placed on ice in individual sealed plastic bags to limit the loss of ectoparasites, and dissected within 48 hours of capture. We measured the total length (TL), weight, and gutted weight for each fish. We examined the oral cavity, skin, fins, muscle tissue, and internal organs of each individual host using a dissecting microscope. Parasites were fixed in 4% hot formalin or 70% ethanol, and mounted for identification following Vidal-Martínez et al. [33]. Adult digeneans were stained with hydrochloric carmine, and mounted in Canada balsam. Nematodes were cleared using increasing concentrations of glycerin, and were temporarily mounted on glass slides. Monogeneans were stained with Gomori's trichrome and mounted in Canada balsam to study their internal structures; we also mounted unstained individuals in ammonium pictrate to study their sclerotized parts. Isopods were identified using descriptions provided in a field guide for Caribbean isopods [34]. The digenean *Lecithochirium floridense* was identified based on descriptions from a published record of parasitism in Caribbean lionfish [30]. There are few published records of lionfish parasites in the Caribbean [30–32], thus we consulted taxonomic specialists to aid with the identification. Trematodes and nematodes were identified by M.L. Aguirre-Macedo, V. Vidal-Martínez, and D. González-Solís. Monogeneans were identified by E. Mendoza-Franco. Two unidentified parasites, an adult trematode and an encysted trematode metacercaria, were only encountered once.

For each site, parasite data consisted of parasite species richness (number of parasite species infecting lionfish captured at each site) and abundance (mean number of individual parasites in infected and uninfected lionfish). Since the parasite data did not meet the assumptions of parametric tests, we used generalized linear models (GLM) with a Poisson error distribution to examine the possible relationship between latitude and parasite richness and abundance. Because preliminary analyses revealed significant differences in lionfish size across sites (ANOVA: F_1,269_ = 10.67, P = 0.0012), we included TL of the host as a covariate in the analyses. For each measure of parasitism we compared the null and expanded models using the Akaike's Information Criterion corrected for small sample sizes (AIC_c_). Models for which AIC_c_s differed from the lowest score by less than two were considered to explain the data equally well [35]. Analyses were done both with and without data for Galeta Point because, as we described above, it constituted a different type of habitat.

To examine how differences in sampling effort (Table 1) may affect our results, we plotted parasite species accumulation curves (observed and expected) for each site grouped by region (i.e. Florida, Yucatan, Belize, Panama). Additionally, we randomly re-sampled 17 observations (the smallest collection effort in our survey-Table 1) from each site 999 times with replacement. For each iteration, a GLM with a Poisson error distribution estimated the effect of latitude on parasite richness. This technique revealed that significant associations between latitude and parasitism were consistent 98% of the time for ectoparasite richness, and 100% of the time for ectoparasite abundance, indicating that differences in sampling effort did not influence our results.

To account for the relative abundance of parasite species, we calculated Shannon's and inverse Simpson's diversity indices for each site. We then used a regression analysis to examine the association between latitude and parasite diversity.

At each site, we calculated host condition for each individual lionfish (infected and uninfected) as the residual of a linear regression between log-transformed TL and gutted weight. We then used regression analysis to determine if there was an association between parasite abundance and host condition at any given site. We used this approach as an initial step to determine whether parasites had a measurable effect on lionfish. We could not find a published weight-length relationship value for lionfish (or the native species considered in the following comparison), and as a result we could not account for natural variability in weight and length. Instead, we examined how host condition varies as a function of parasite abundance.

### Comparison of parasites in lionfish and two ecologically similar native species

To begin to compare parasitism between lionfish and native species, we collected *P*. *volitans* and two ecologically similar native species the graysby (*C*. *cruentata*), and the lizardfish (*S*. *intermedius*) across three sites in Panama, the southernmost region. We focused on sites in Panama for two reasons: (1) we expected higher levels of parasitism in Panama based on results of our regional survey (first component of this study); and (2) for logistical reasons given proximity to our facilities. Native species were selected based on ecological characteristics (e.g. diet, behavior, habitat use), abundance in study sites, and published records of interactions with lionfish. For example, the graysby is a mesopredator which shares prey with lionfish [36, 37], they commonly co-occur with lionfish on reefs (personal observation), and, like other mesopredators, may compete with lionfish [28, 29]. We selected *S*. *intermedius* due to similarities in diet with lionfish [36, 37], its behavior, and co-occurrence in sites where we collected lionfish (personal observation).

We collected fish at two sites in Bocas del Toro (Cristobal: 9.30°N, 82.29°W and Hospital Point: 9.33°N, 82.22°W) in Aug- and Sep-2013; and one site in Portobelo (9.55°N, 79.68°W) in Nov- and Dec-2013. The three sites were characterized by fringing coral reefs and ranged in depth from 12–20 m. Differences in the abundance of each species resulted in differences in the collection effort across sites (S1 Table). The methodology to collect and examine fish in this comparison was the same as above with one exception. Due to the large number of monogeneans found on *C*. *cruentata*, we rinsed the skin of the fish in fresh water to remove skin parasites, filtered the water using a 60 μm mesh, and examined the mesh using a dissecting microscope. The same approach was used for all host species collected for this comparison.

Because we were unable to collect all four species at every site (S1 Table), we compared each native species to *P*. *volitans* independently. To compare parasite abundance and richness in *C*. *cruentata* and *P*. *volitans* across the three sites, we used a generalized linear mixed model with a Poisson error distribution in which host species was treated as a fixed factor and site as a random factor. Given the absence of *S*. *intermedius* in all but one site, we used a non-parametric unpaired Wilcoxon's Rank Sum test to compare parasite richness and abundance. To address potential bias resulting from differences in our sampling effort across host species, we plotted parasite species accumulation curves for each host at each site. We calculated host condition as above and examined the association between parasite abundance and individual condition by host species.

All analyses were carried out with R version 3.0.2. We used package 'vegan' to calculate diversity indices and plot accumulation curves. We fitted the generalized mixed effects models using the 'glmer' function from package 'lme4'.

## Results

### Regional comparison of lionfish parasites

**Fig 1:**
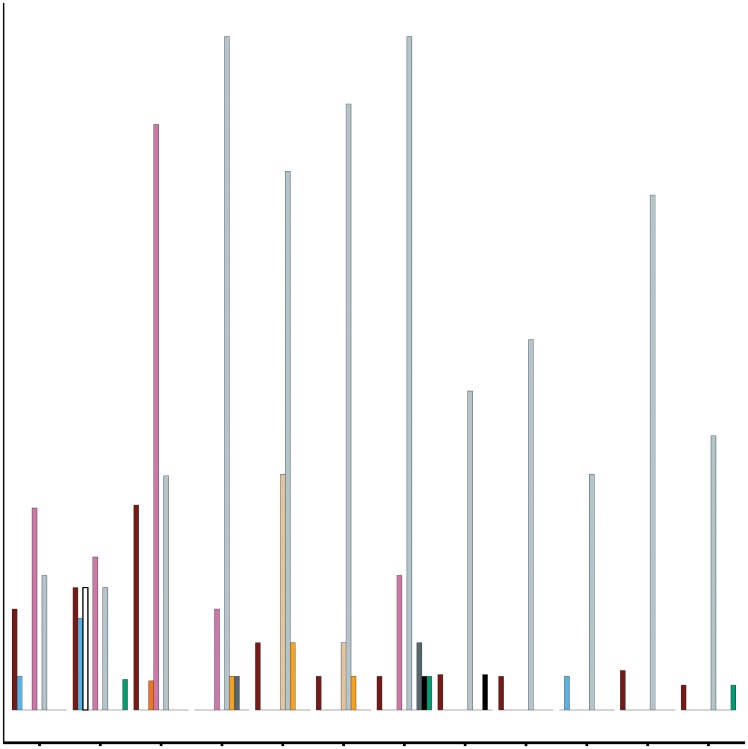
Infection prevalence (% host infected) for all parasite taxa found infecting *P*. *volitans*. Each taxon is identified by a specific color (see key). Latitude increases from left to right, and all sites are shown, except for the outlying back reef site in Galeta Point. Sample size (hosts examined) at each site ranged from 17–27; see Table 1 for details on collection effort. Species in legend with asterisk represent a new record for *P*. *volitans*.

**Fig 2:**
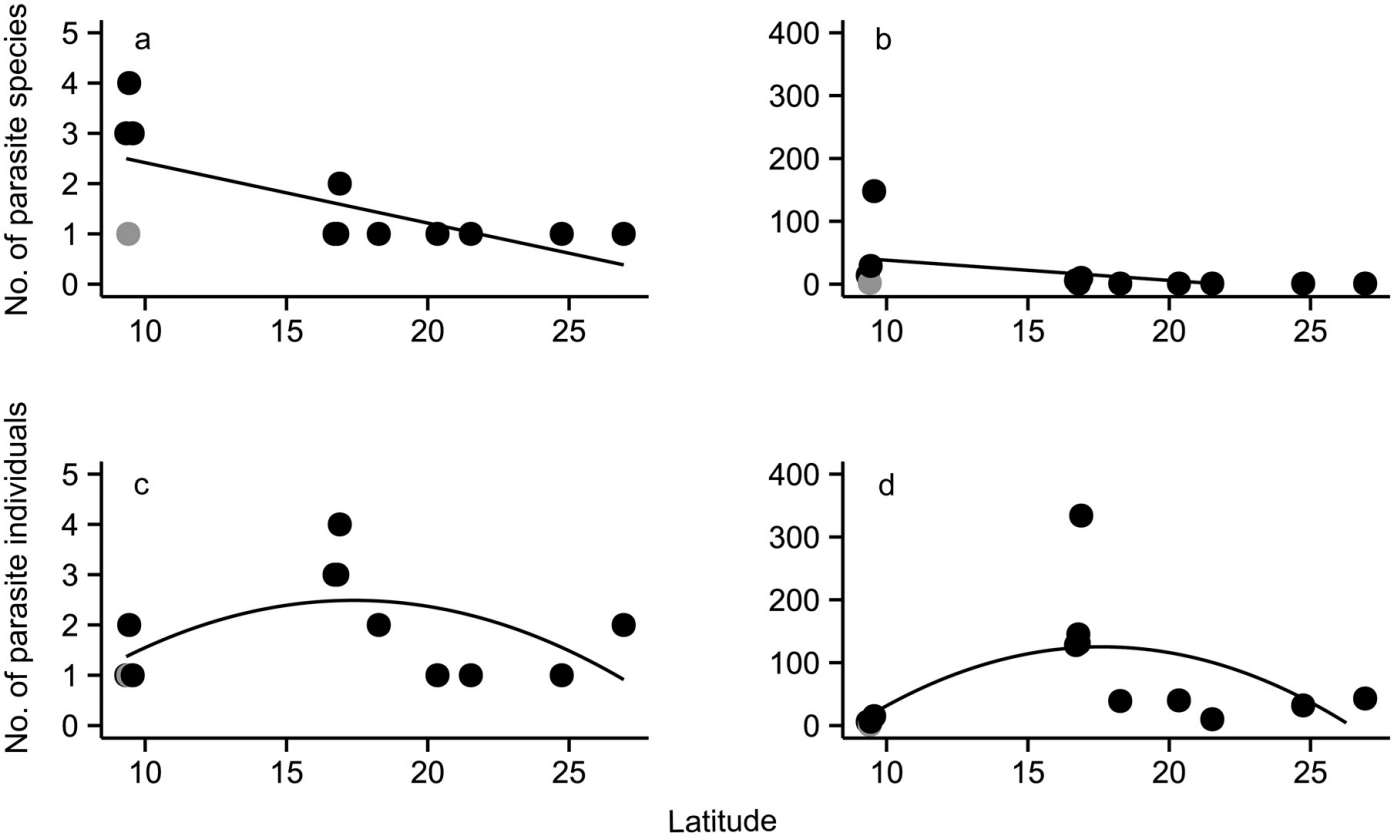
Species richness and abundance of parasites infecting *Pterois volitans* as a function of latitude. Each point represents the total species richness (number of species) of ectoparasites (A) and endoparasites (B), or abundance (number of individuals) of ectoparasites (C) and endoparasites (D) infecting *Pterois volitans* at a given site. The point for Galeta Point (the outlying back-reef site) is shown in grey. Latitude had a significant negative effect on ectoparasite richness (P = 0.028) and abundance (P<0.0001) when Galeta Point was removed from analysis. Endoparasite abundance and richness were not associated with latitude. Sample size (hosts examined) at each site ranged from 17–27; see Table 1 for details on collection effort.

We encountered twelve parasite species infecting lionfish, including trematodes, nematodes, monogeneans, turbellarians, and isopods (Fig 1). Overall, endoparasite richness was highest in Belize, while ectoparasite richness was highest in Panama. Ectoparasite species richness and abundance increased on lionfish with decreasing latitude (Fig 2). The model that best described patterns in ectoparasite richness and abundance accounted solely for the effect of latitude (S2 Table); that model showed that latitude had a small yet significant negative effect on both measures for ectoparasites (ectoparasite richness: coefficient = -0.095, P = 0.028; ectoparasite abundance: coef. = -0.34, P<0.0001). This effect was significant only when the inner lagoon site (Galeta, Panama) was removed from the analysis (S2 Table). Ectoparasite abundance in Portobelo, Panama was more than five times greater than in any other site surveyed (Fig 2), and may strongly influence the observed patterns. While the effect of latitude on ectoparasite abundance was weaker when data from Portobelo were removed from the analysis, the effect was still significant (coef. = -0.22, P<0.0001).

**Fig 3:**
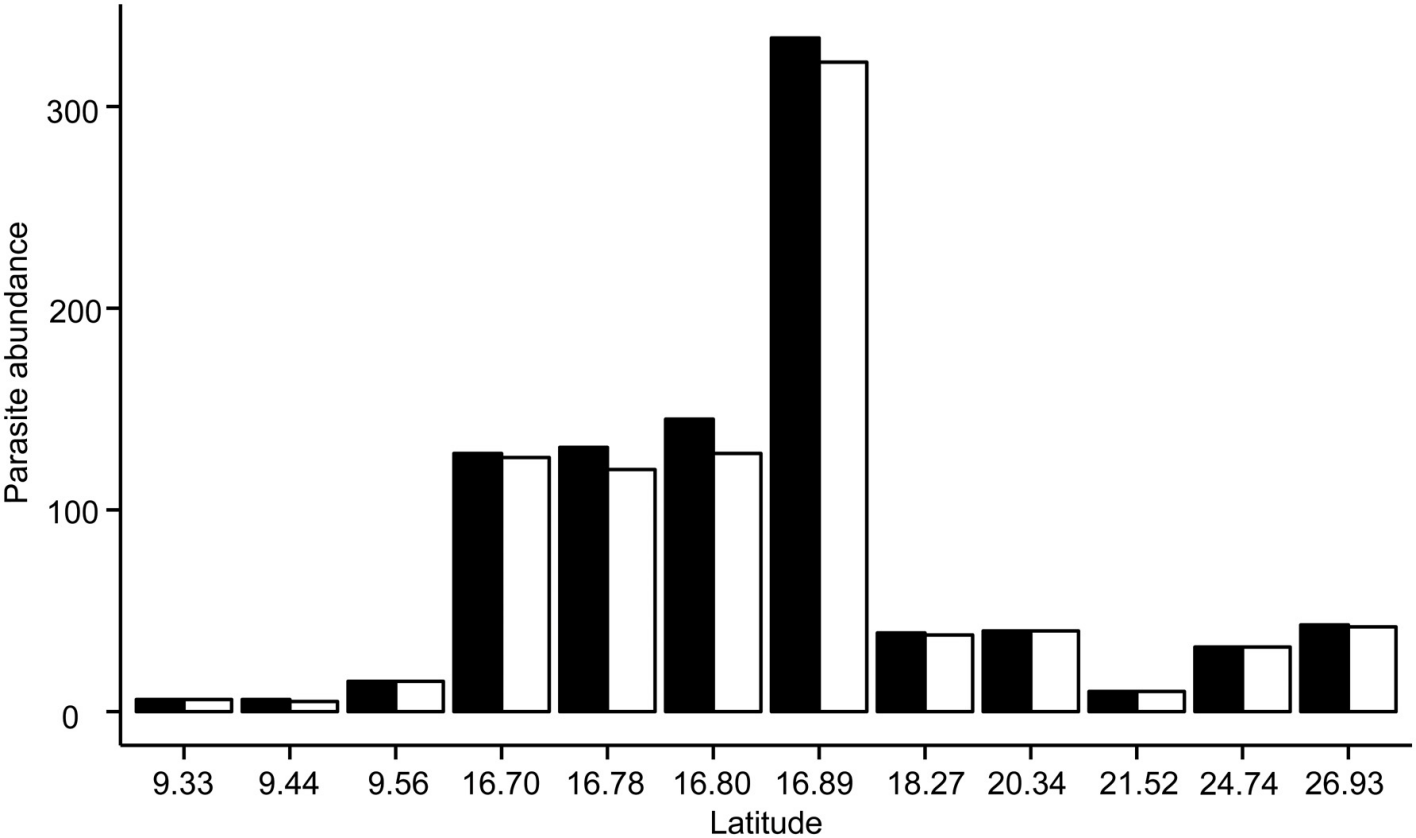
Abundance of all endoparasites, and *Lecithochirium floridense* in *P*. *volitans* by latitude. Black bars represent endoparasite abundance (total number of parasites in infected and uninfected hosts), while white bars represent abundance of *L*. *floridense*. Latitude increases from left to right, and all sites, except for the outlying back reef site in Galeta Point, are shown. Sample sizes (hosts examined) at each site ranged from 17–27; see Table 1 for details on collection effort.

Endoparasite richness varied across regions, but was neither associated with latitude nor host length, and peaked in sites in Belize (S2 Table, Fig 2), where lionfish were infected with over twice as many endoparasite species as lionfish from other regions. Endoparasite abundance was also highest in Belize (Fig 2), and was best predicted by the model accounting solely for TL (S2 Table). Differences in endoparasite abundance across sites were driven primarily by the trematode *L*. *floridense* which infected lionfish at all sites (Fig 3), and infected 80–100% of the hosts in Belize (Fig 1).

**Fig 4:**
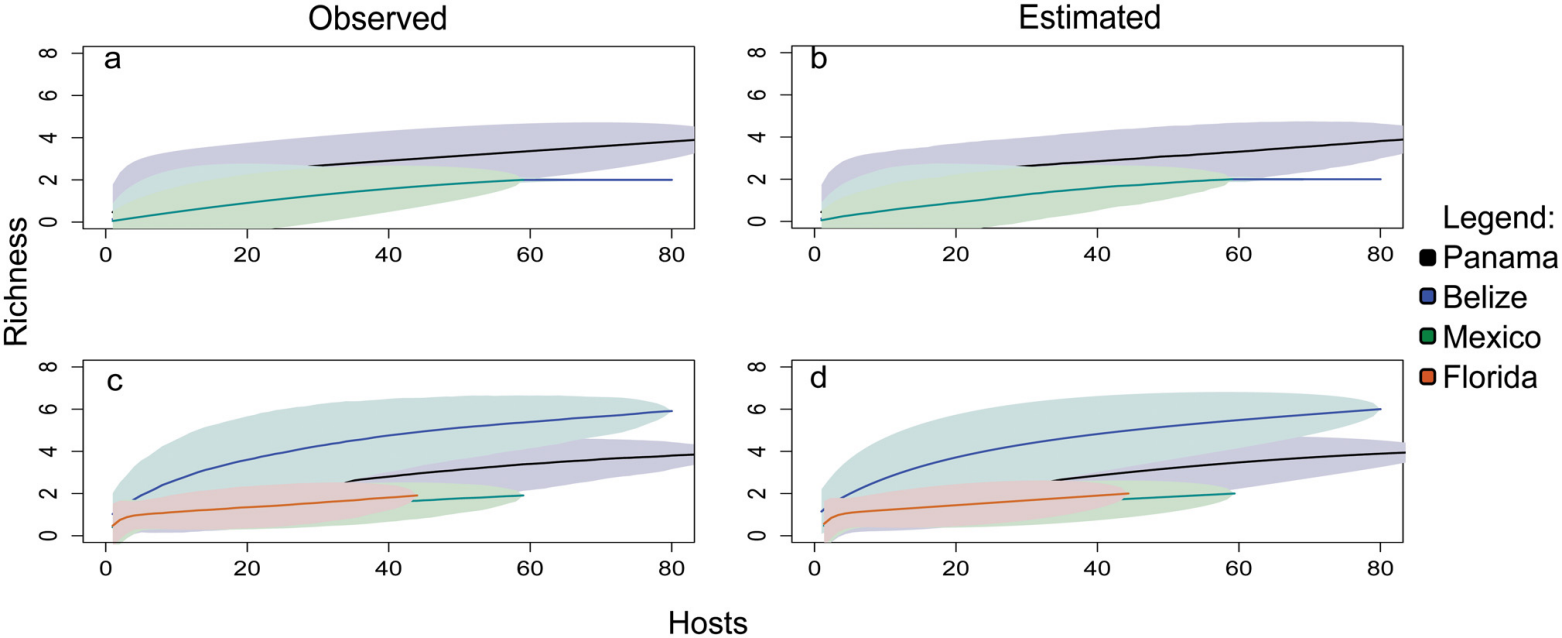
Parasite species accumulation curve for sites grouped by region. Accumulation curves for ectoparasites based on observed richness and estimated richness are shown in (A) and (B), respectively, and for endoparasites in (C) and (D). Each color represents a specific region: Panama (black), Belize (blue), Yucatan (green), Florida (orange). No curve was plotted for Florida in (a) because only one ectoparasite taxon infected *P*. *volitans* collected at those sites.

The species accumulation curves demonstrate that the latitudinal patterns in ectoparasite richness were not affected by differences in sample size across sites (Fig 4). Accumulation curves for ectoparasites in Belize and Yucatan reached an asymptote, while those for sites in Panama continued to marginally increase (Fig 4). None of the endoparasite species accumulation curves reached an asymptote, and continued to increase slightly (Fig 4).

Across all sites, the prevalence of most individual parasite species was less than 25%, except for the trematode *L*. *floridense* and an unidentified gill turbellarian which exceeded 50% at some sites (Fig 1). Over a third of the parasite species were represented by only one individual at a given site. Thus, to account for rare species, we used Shannon's and inverse Simpson's diversity indices to examine the effect of latitude on parasite diversity. Consistent with above, ectoparasite diversity was significantly negatively correlated with latitude (Simpson's: r^2^ = 0.37, P = 0.021; Inv. Shannon's: r^2^ = 0.44, P = 0.011), while endoparasite diversity was not correlated with latitude (Simpson's: r^2^ = 0.033, P = 0.27; Inv. Shannon's: r^2^ = 0.024, P = 0.41).

Overall, parasite abundance was not associated with host condition with the exception of one site, Naranjo Island, Panama. While ectoparasite abundance was weakly associated with host condition at that site (r^2^ = 0.18, P = 0.029), endoparasite abundance was not associated with host condition at any site (S3 Table).

### Comparison of parasites in lionfish and two ecologically similar native species

**Fig 5:**
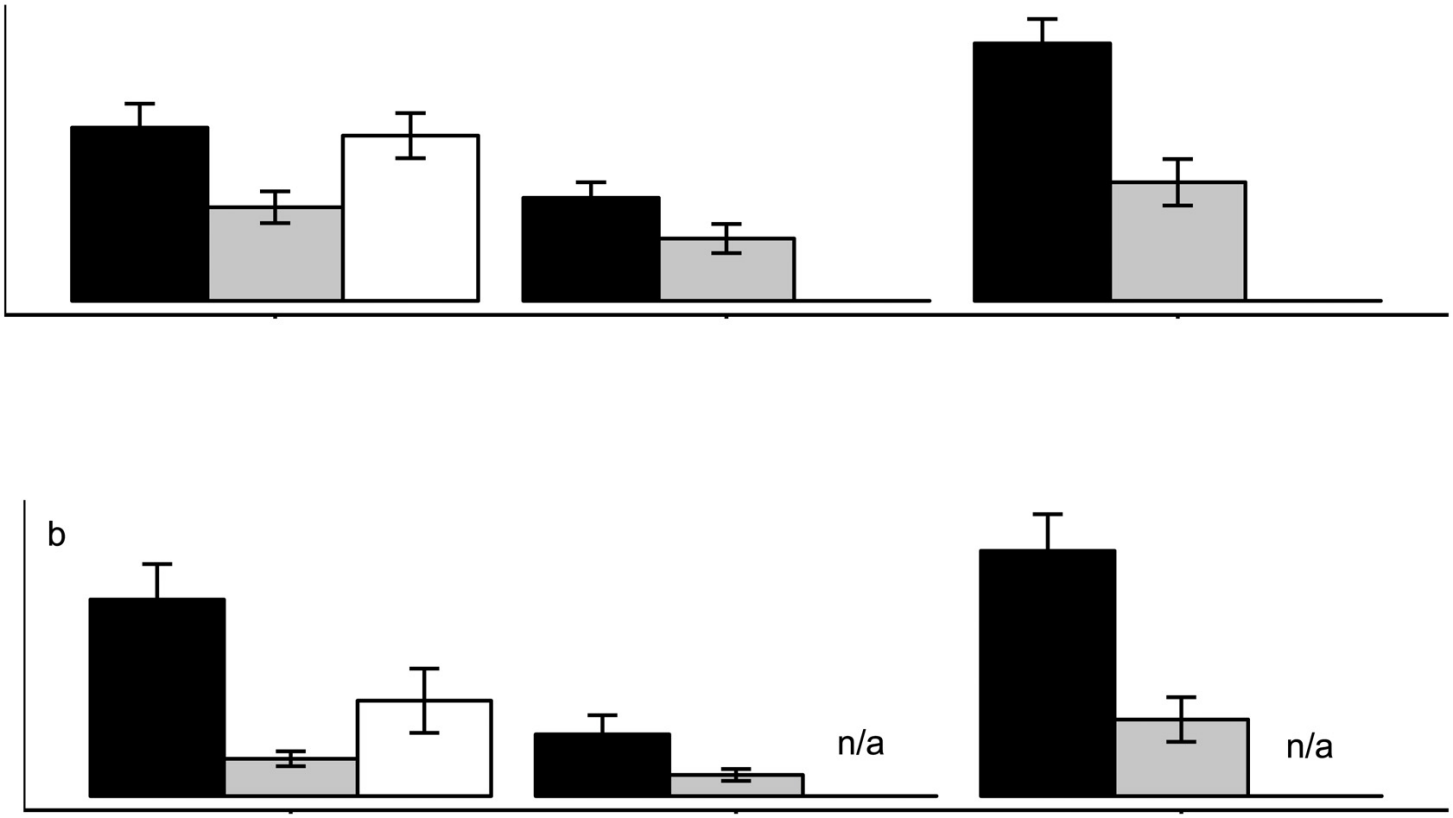
Parasite richness and abundance for lionfish and native hosts in three sites in Panama. Parasite richness (mean number of parasite taxa per host ± SE) is shown in (A), while parasite abundance (mean number of individual parasites per host ± SE) is shown in (B). Each host species is represented by a specific color: *Cephalopholis cruentata* (black bars), *P*. *volitans* (grey bars), and *Synodus intermedius* (white bars). Sample sizes for each species varied among sites: Cristobal (*C*. *cruentata*, n = 19; *P*. *volitans*, n = 19; *S*. *intermedius*, n = 14), Hospital Point (*C*. *cruentata*, n = 20; *P*. *volitans*, n = 17), and Portobelo (*C*. *cruentata*, n = 19; *P*. *volitans*, n = 21). Note that *S*. *intermedius* was collected only in Cristobal and was absent in the other sites.

**Fig 6:**
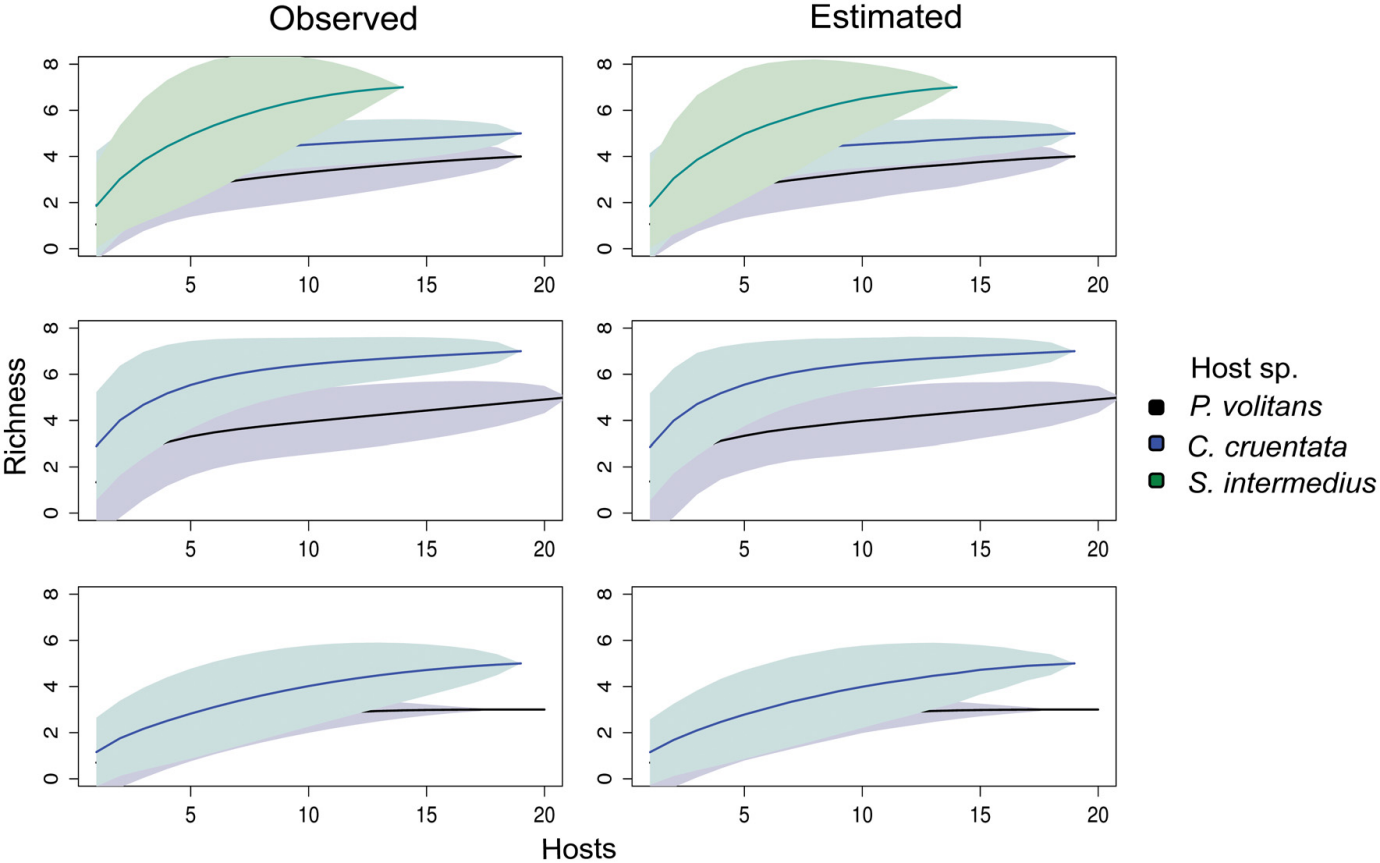
Parasite species accumulation curves for lionfish and native hosts collected in three sites in Panama. The species accumulation curves based on observed and expected richness for Cristobal are shown in (A) and (B), respectively, for Portobelo in (C) and (D), and for Hospital Point in (E) and (F). Each host species is represented by a specific color: *P*. *volitans* (black), *C*. *cruentata* (blue), and *S*. *intermedius* (green). Note that *S*. *intermedius* was collected only in Cristobal and is therefore absent from the other sites.

Parasite species richness and abundance were significantly higher in the native graysby (*C*. *cruentata*) than in lionfish (Richness: mean = 0.98, P = 0.00016; Abundance: mean = 9.01, P<0.0001); this pattern was consistent across all sites in Panama (Fig 5). Similarly, species richness and abundance were significantly higher in *S*. *intermedius* compared to lionfish in Cristobal, where the two host species overlap (Richness: Wilcoxon Rank Sum test—mean = 0.80, W = 75, P = 0.026; Abundance: Wilcoxon Rank Sum test—mean = 4.25, W = 79.5, P = 0.049; Fig 5). Species accumulation curves confirmed that the native *C*. *cruentata* and *S*. *intermedius* were infected with a greater number of parasite species than lionfish, irrespective of differences in sampling effort (Fig 6). Even though there were significant differences in parasite richness between lionfish and native fishes, those differences were relatively small (Fig 5a).

Three parasite species infected both lionfish and at least one of the native fish species. These parasites included the monogenean *Neobenedenia* sp. (infected *P*. *volitans* and *C*. *cruentata*), gnathiid isopods (infected all hosts), and an unidentified gill turbellarian (infected *P*. *volitans* and *C*. *cruentata*). The abundance and prevalence of these shared parasites varied among host species (see S4 Table for list of shared parasites).

Parasite abundance was significantly negatively associated with the condition of *C*. *cruentata* (r^2^ = 0.20, P = 0.00034), but not *P*. *volitans* (r^2^ = 0.023, P = 0.13) or *S*. *intermedius* (r^2^ = 0.05, P = 0.55).

## Discussion

### Regional comparison of lionfish parasites

The rapid and widespread invasion of the Indo-pacific lionfish, *P*. *volitans*, provides an opportunity to test how native parasites are accumulated by introduced species across a broad spatial scale. We found different patterns of parasitism for endo- and ectoparasites across the four regions that we surveyed in the western Atlantic. Ectoparasite species richness and abundance increased in lionfish from Florida to Panama, whereas endoparasite species richness and abundance were not linearly associated with latitude. Instead, lionfish from Belize were infected with over twice as many endoparasite species than other regions, and endoparasite abundance was also highest in Belize, driven primarily by the native trematode *L*. *floridense*. Our findings are consistent with Rohde and Heap [38] who found that species richness and abundance of ectoparasites, but not of endoparasites of native marine fishes, were higher at low latitudes. While we did detect a significant increase in ectoparasite species richness with decreasing latitude, we caution that only examining four general regions limits the ability to detect any potential latitudinal gradient.

Twenty seven parasite species have been reported to date for lionfish in the Western Atlantic [32], and, here, we report six additional species (indicated by asterisks in Fig 1), bringing the total number thus far to thirty three for the region. Ramos-Ascherl et al. [32] report higher parasite species richness (mostly endoparasites) in the Bahamas, Puerto Rico and Cayman Islands than we report in this study underscoring the importance of regional differences in parasite accumulation in the introduced range. However, differences in sampling methods and spatial scales between the two studies make direct comparisons difficult. Ramos-Ascherl et al. [32] collected 326 lionfish across three northern regions from a variety of habitat types, while we examined 271 lionfish from four regions across 17 degrees of latitude. Thus, it is possible (if not likely) that parasite richness scales with number of habitat types sampled, increasing the relative richness in the earlier study compared to ours. Alternatively, the available data may reflect real differences in parasitism across regions. A survey of parasitism on lionfish across latitudes along the eastern edge of the Caribbean, controlling for habitat type(s), would prove useful to resolve and interpret these regional patterns.

Endoparasite richness and abundance were not linearly associated with latitude among our study sites, and may instead be driven, at least in part, by site-specific differences in lionfish size and abundance. Host size is known to be a good predictor of parasite richness, and parasitism tends to increase with host size [21]. Lionfish collected in Belize, where endoparasite richness and abundance were highest, were significantly larger than those from other regions, and our results indicate that fish length had a significant effect on endoparasite abundance. In addition, many of the endoparasites that we encountered were adult stages of trophically transmitted parasites. Thus, patterns of parasitism in intermediate hosts combined with lionfish diet breath could influence the pattern we observed, however, further research is needed to examine the association between prey diversity and endoparasites in lionfish.

Site variability in endoparasite abundance was driven largely by the trematode *L*. *floridense*, particularly in Belize. High *L*. *floridense* abundances in Belize could be due to lionfish consuming shrimp infected by the metacercarial cyst stage of this trematode (AJS and MET personal observation). In Belize, we observed that the stomachs of lionfish with high *L*. *floridense* intensities were also filled with small unidentified shrimp which were heavily infected with metacercaria. *L*. *floridense* is reported to be a generalist parasite that, in its adult stage, infects multiple fish species [39–41], yet its intermediate hosts have not been identified. The shrimp infected by metacecarial cysts that we observed in lionfish stomachs were partially digested and we were unable to identify this potential intermediate host. The numbers of shrimp in the stomachs of lionfish from Belize were higher relative to our other study sites, suggesting that higher consumption of infected prey in this region may have led to the high abundance of *L*. *floridense*.

Introduced species can accumulate parasites as a function of time since establishment, and parasite richness in introduced populations can increase over time, independent of invader abundance and individual size (age)[42, 43]. While the association between time since establishment and parasitism in introduced hosts can explain a significant proportion of the variance in parasite richness among populations of an invader [43], we do not believe this to be the case for lionfish. Lionfish spread rapidly through the Western Atlantic, and invaded the regions where our surveys took place over a three year interval [27]. Thus, the lionfish's rapid spread should minimize the effect of time on parasite accumulation across the western Atlantic. However, we cannot fully exclude the possibility that time has played some role and interacts with geography to shape observed patterns of parasitism.

Our results suggest there is currently a limited direct effect of parasites on the lionfish populations. Indeed, over one third of the parasite species we found in lionfish were represented by a single individual at any given site, and prevalence of most parasite species was below 25%. Further, we found no coarse-level association between lionfish condition and parasite abundance, however we note this could also reflect a lack of sensitivity of the metric, or that parasites often affect hosts in other ways (e.g. survival, reproduction).

### Comparison of parasites in lionfish and two ecologically similar native species

Parasites can influence interactions between competing hosts through density- and trait-mediated effects [44]. Introduced hosts often escape parasites present in their native range, and accumulate fewer novel parasites in their exotic range [5, 16]. As a result, native hosts may be more heavily infected by parasites than introduced competitors, which can translate into benefits for the invader [15]. As an initial step in evaluating how differential parasitism could influence competitive interactions between lionfish and native species, we compared parasitism between lionfish and two ecologically similar native hosts. Parasite species richness was nearly two times higher in the graysby grouper *Cephalopholis cruentata*, and the lizardfish, *Synodus intermedius* compared to lionfish in the southernmost region, Panama. Similarly, parasite abundance was more than three times higher in the native *C*. *cruentata* compared to lionfish. Further, we found that parasite abundance had a significant negative effect on the condition of *C*. *cruentata*, but not *P*. *volitans* or *S*. *intermedius*. While further research is needed to substantiate these results, the pattern is consistent with a recent study indicating that native cichlids were more heavily parasitized compared to introduced cichlids, and that parasites had a significant negative effect on native fish condition but not on the invader [45].

Three parasites infected both the native fishes and the introduced lionfish, including a gnathiid isopod, a monogenean, *Neobenedenia* sp., and an unidentified gill turbellarian. Gnathiid isopods are common generalist parasites of marine fish [46], and have previously been reported from lionfish in the Caribbean [47]. Consistent with our results, Sikkel et al. [47] found higher gnathiid isopod prevalence and intensity on native fishes compared to lionfish; however, they report higher gnathiid isopod intensities than in our study. Gnathiid isopods are most abundant on hosts at night and dawn [48–49], thus differences between our results may reflect variation in the time at which lionfish were collected. Our collections took place during the day, between 07:00 and 17:30, while Sikkel et al. [47] examined lionfish collected at dawn, when gnathiids are thought to be most active. The monogenean *Neobenedenia* sp. infected both lionfish and *C*. *cruentata* and parasites within this genus are known to infect a wide variety of hosts belonging to a range of families [50].

## Conclusion

Together with previous studies [30–32], our results suggest that lionfish are accumulating native parasites across their introduced range and patterns of parasitism vary regionally. Specifically, our results indicate that ectoparasite species richness was highest in Panama, the southernmost region sampled, whereas endoparasites were more diverse in Belize. Although lionfish accumulated new parasites in their introduced range, the absence of an association between lionfish condition and parasite abundance suggests that parasites probably do not have a substantial direct effect on the invader, but studies examining more subtle effects of parasites are warranted. Parasites may also have indirect effects on lionfish. For example, native mesopredatory fish, like the graysby, may compete with lionfish [28], and differential parasitism could influence competitive interactions between lionfish and native hosts if native hosts suffer disproportionately from the effects of parasitism. In Panama both the graysby and the lizzardfish were more heavily parasitized (in terms of richness and abundance) than lionfish, suggesting that parasites could differentially impact the native species and the invader. While recent studies suggest that lionfish probably compete with these native mesopredators [28, 29] further research is needed to examine how parasites could alter these competitive interactions and influence the invasion of lionfish across it's expanding range in the western Atlantic.

## Supporting Information

S1 Table Summary of collection effort for parasitological comparison between native hosts and *Pterois volitans*. Species labeled 'Native' are native to the Caribbean, while *P*. *volitans* was introduced from the Indo-Pacific.
(PDF)

S2 Table Ranking of generalized linear models for the effect of latitude (Lat) and host length (TL) on parasite (ecto- and endo-) richness and abundance using Akaike's Information Criterion corrected for small sample sizes (AIC_c_). The table is grouped by parasite group and measure of parasitism (i.e. ecto- and endoparasite richness and abundance). Values under the 'With Galeta Point' columns outline results from models using the complete data set, while values under the 'Without Galeta Point' show results from models using the data set without the outlying observations made at that site. Models for which AIC_c_s differed from the lowest score by less than two are considered to explain the data equally well. The most parsimonious model for a particular measure of parasitism is indicated by values in bold. R^2^ represent McFaddens's Pseudo-R^2^ values.
(PDF)

S3 Table Results for regressions between *Pterois volitans* individual condition and parasite abundance across latitude. Values in bold indicate a significant effect of parasite abundance on host condition.
(PDF)

S4 Table Abundance and percent prevalence (% host infected) of parasites infecting introduced *Pterois volitans* and native species examined in the present survey (*Cephalopholis cruentata* and *Synodus intermedius*) at three sites in Panama. (PDF)

S5 Table Original data for regional survey of *Pterois volitans* metazoan parasites in the Western Atlantic. (XLSX)

S6 Table Original data for comparison of parasitism between *Pterois volitans* and ecologically similar native species (*Cephalopholis cruentata* and *Synodus intermedius*) at three sites in Panama. (XLSX)
