# Supplementary material for: Regional Variation in Parasite Species Richness and Abundance in the Introduced Range of the Invasive Lionfish, Pterois volitans
Source: PLoS One. 2015 Jun 22;10(6):e0131075. doi: 10.1371/journal.pone.0131075 (PMC4476800; doi:10.1371/journal.pone.0131075)
Supplement: S1 Table — Species labeled 'Native' are native to the Caribbean, while P. volitans was introduced from the Indo-Pacific. (PDF) [file pone.0131075.s001.pdf]

S1 Table. Summary of collection effort for parasitological comparison between native hosts and *Pterois volitans.* Species labeled 'Native' are native to the Caribbean, while *P. volitans* was introduced from the Indo-Pacific.

Host species	Native/Introduced	Cristobal	Hospital Point	Portobelo
Pterois volitans	Introduced	19	17	21
Cephalopholis cruentata	Native	19	20	19
Synodus intermedius	Native	14	0	0
