# Supplementary material for: Regional Variation in Parasite Species Richness and Abundance in the Introduced Range of the Invasive Lionfish, Pterois volitans
Source: PLoS One. 2015 Jun 22;10(6):e0131075. doi: 10.1371/journal.pone.0131075 (PMC4476800; doi:10.1371/journal.pone.0131075)
Supplement: S2 Table — The table is grouped by parasite group and measure of parasitism (i.e. ecto- and endoparasite richness and abundance). Values under the 'With Galeta Point' columns outline results from models using the complete data set, while values under the 'Without Galeta Point' show results from models using the data set without the outlying observations made at that site. Models for which AICcs differed from the lowest score by less than two are considered to explain the data equally well. The most parsimonious model for a particular measure of parasitism is indicated by values in bold. R2 represent McFaddens's Pseudo-R2 values. (PDF) [file pone.0131075.s002.pdf]

S2 Table. Ranking of generalized linear models for the effect of latitude (Lat) and host length (TL) on parasite (ecto- and endo-) richness and abundance using Akaike's Information Criterion corrected for small sample sizes (AIC_c). The table is grouped by parasite group and measure of parasitism (i.e. ecto- and endoparasite richness and abundance). Values under the 'With Galeta Point' columns outline results from models using the complete data set, while values under the 'Without Galeta Point' show results from models using the data set without the outlying observations made at that site. Models for which AIC_cs differed from the lowest score by less than two are considered to explain the data equally well. The most parsimonious model for a particular measure of parasitism is indicated by values in bold. R^2 represent McFaddens's Pseudo-R^2 values.

			With Galeta Point				Without Galeta Point			
	Model	# parameters	logLik	AICc	ΔAICc	R^2	logLik	AICc	ΔAICc	R^2
Ectoparasite Richness	Null	1	**-18.15**	**38.66**	**1.27**	**-**	-17.03	36.46	2.41	-
	Lat	2	-16.09	37.39	0.00	0.11	**-14.36**	**34.05**	**0.00**	0.15
	TL	2	-17.53	40.26	2.87	0.03	-15.63	36.60	2.55	0.05
	Lat+TL	3	-16.06	40.80	3.41	0.11	-13.80	36.60	2.55	0.16
Ectoparasite Abundance	Null	1	-320.68	643.72	415.55	-	-308.40	619.20	385.96	-
	Lat	2	**-131.48**	**228.17**	**0.00**	**0.49**	**-113.95**	**233.24**	**0.00**	**0.63**
	TL	2	-296.02	597.24	369.07	0.08	-244.26	493.85	260.61	0.21
	Lat+TL	3	-169.85	388.37	160.20	0.56	-113.92	236.84	3.60	0.63
Endoparasite Richness	Null	1	**-19.28**	**40.93**	**0.00**	**-**	**-18.00**	**38.39**	**0.00**	**-**
	Lat	2	-19.26	43.73	2.80	0.00	-18.00	41.32	2.93	0.00
	TL	2	-18.18	41.56	0.63	0.06	-17.18	39.69	1.30	0.05
	Lat+TL	3	-18.16	44.98	4.05	0.06	-17.15	43.31	4.92	0.05
Endoparasite Abundance	Null	1	-633.08	1268.52	672.78	-	-562.99	1128.38	541.53	-
	Lat	2	-620.65	1246.49	650.75	0.02	-561.27	1127.88	541.03	0.00
	TL	2	**-295.27**	**595.74**	**0.00**	**0.53**	**-290.76**	**586.85**	**0.00**	**0.48**
	Lat+TL	3	-294.81	598.28	2.54	0.53	-289.99	588.98	2.13	0.49
