# Supplementary material for: Regional Variation in Parasite Species Richness and Abundance in the Introduced Range of the Invasive Lionfish, Pterois volitans
Source: PLoS One. 2015 Jun 22;10(6):e0131075. doi: 10.1371/journal.pone.0131075 (PMC4476800; doi:10.1371/journal.pone.0131075)
Supplement: S3 Table — Values in bold indicate a significant effect of parasite abundance on host condition. (PDF) [file pone.0131075.s003.pdf]

S3 Table. Results for regressions between *Pterois volitans* individual condition and parasite abundance across latitude. Values in bold indicate a significant effect of parasite abundance on host condition.

	Ectoparasites			Endoparasites		
Latitude	Coefficient	r^2	P	Coefficient	r^2	P
9.332	0.012	0.12	0.13	-0.013	0.069	0.26
9.404	0.032	0.057	0.27	-0.022	0.013	0.60
9.435	**-0.010**	**0.22**	**0.029**	-0.0022	0.00088	0.90
9.559	0.00034	0.0034	0.79	-0.0015	0.0039	0.78
16.701	-0.0065	0.053	0.33	-0.0017	0.17	0.073
16.784	-0.11	0.080	0.23	0.00076	0.0010	0.89
16.798	0.062	0.19	0.057	-0.0013	0.035	0.43
16.886	0.0022	0.004	0.79	-0.00033	0.018	0.58
18.266	-0.014	0.0027	0.83	0.0016	0.0044	0.78
20.343	0.044	0.057	0.31	-0.0055	0.15	0.089
21.524	-0.039	0.016	0.60	-0.0012	0.00018	0.96
24.741	0.020	0.033	0.48	0.0053	0.11	0.19
26.932	-0.0097	0.0015	0.85	-0.0037	0.035	0.36
