# Supplementary material for: Regional Variation in Parasite Species Richness and Abundance in the Introduced Range of the Invasive Lionfish, Pterois volitans
Source: PLoS One. 2015 Jun 22;10(6):e0131075. doi: 10.1371/journal.pone.0131075 (PMC4476800; doi:10.1371/journal.pone.0131075)
Supplement: S4 Table — (PDF) [file pone.0131075.s004.pdf]

S4 Table. Abundance and percent prevalence (% host infected) of parasites infecting introduced *Pterois volitans* and native species examined in the present survey (*Cephalopholis cruentata* and *Synodus intermedius*) at three sites in Panama.

		Neobenedenia sp.		*Gnathiia* sp.		Turbellaria	
Site	Host species	Abundance	%Prev	Abundance	%Prev	Abundance	%Prev
Cristobal	*P. volitans*	0.05	5.00	1.10	36.84	0.95	52.63
	C. cruentata	0.20	5.26	0.40	26.32	1.20	47.37
	S. intermedius	0.00	0.00	0.57	50.00	0.00	0.00
Hospital Point	*P. volitans*	0.00	0.00	19.00	42.11	0.37	15.79
	C. cruentata	0.00	0.00	2.89	73.68	0.21	10.53
Portobelo	*P. volitans*	0.10	4.76	0.65	33.33	2.00	52.38
	C. cruentata	0.89	31.58	3.37	89.47	0.26	26.32
